# Health, income and poverty: evidence from China’s rural household survey

**DOI:** 10.1186/s12939-020-1121-0

**Published:** 2020-03-17

**Authors:** Yang Zhou, Yuanzhi Guo, Yansui Liu

**Affiliations:** 1 Institute of Geographic Sciences and Natural Resources Research, Chinese Academy of Sciences, Beijing, 100101 China; 2 Key Laboratory of Regional Sustainable Development Modeling, Chinese Academy of Sciences, Beijing, 100101 China; 3 University of Chinese Academy of Sciences, Beijing, 100049 China

**Keywords:** Health status, Poverty alleviation, Health intervention, Poor households, Rural China

## Abstract

**Background:**

Understanding the health status of the poor households and the influence of unhealthy on their income can provide some vital insights into the effectiveness and appropriateness of poverty reduction solutions.

**Methods:**

Based on a nationwide cross-sectional survey of 29,712 rural poor households, this study systematically investigated the causes of poverty and health status of Chinese rural poor households, and revealed the relationship between health, income and poverty.

**Results:**

The health status of the rural poor in China is not optimistic, with 51.63% attributing their poverty to the illness of household members. NCDs are the biggest health threat to the rural poor in China. Over 60% of all the households have at least one patient and more than a quarter of the households with patients cannot afford expensive medical expenses. Although 98% of all the households participate in China’s a rural health insurance system - the New Rural Cooperative Medical Scheme - 16% are still unable to bear their medical expenses after reimbursement from the scheme. Further, high altitude, ill-health and low-income are interlinked and mutually reinforcing. The per capita net income of poor households was inversely proportional to the altitude of their places of residence, family aging and unhealthy status, but was positively correlated with the number of workforces in their families.

**Conclusions:**

Poverty due to illness is one of the root causes of rural poverty in China. With the backward medical infrastructure in high altitude areas, people are more prone to fall into the vicious circle of poverty-unhealthy-low income-poverty. The establishment of effective long-term mechanism of disease prevention and intervention is an important prerequisite to enhance the endogenous development power of the poor and reduce poverty.

## Introduction

**Fig. 1:**
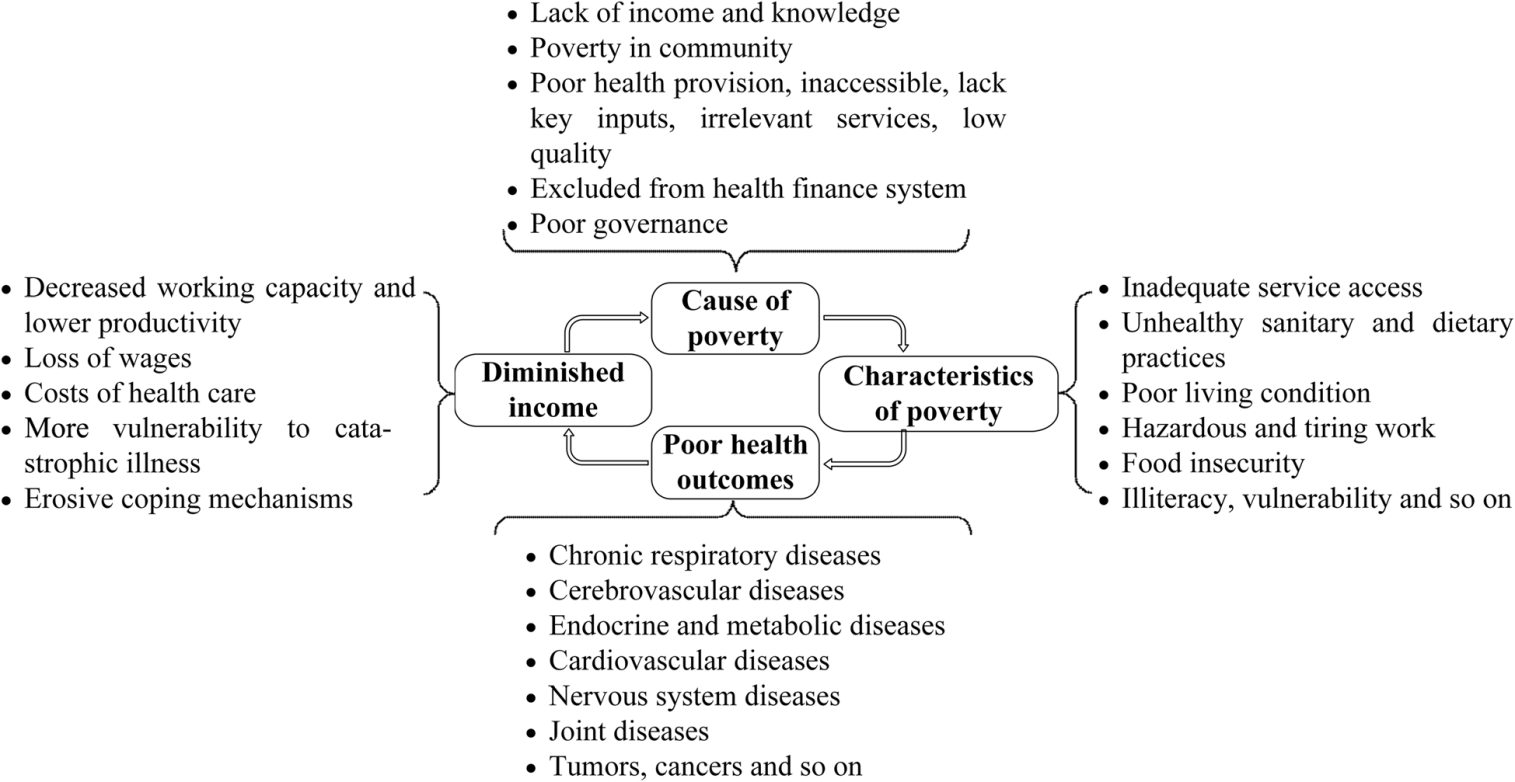
The nexus between poverty and health

Poverty and ill-health are generally believed to be have a bidirectional causality relationship [1–3], and poverty-led diseases disproportionately affect extremely poor populations and contribute to a vicious cycle of poverty because of decreased productivity led by long-term illness and disability (Fig. 1) [4–6]. For the poor, ill-health is said to be fundamental cause of poverty, mainly because poor people are forced to live in unhealthy environments without decent shelter, clean water or adequate sanitation [7–9]. The lack of competent medical personnel in poor communities, and that low-quality care may further compromise health outcome. Additional effects include ill-health being a major factor in the production and reproduction of poverty. Health expenditure often comprises a larger proportion of the incomes of low-income household, increasing their economic burden [10, 11]. Paying for health care has become a notable cause of impoverishment for households [12], and poor quality and ineffective healthcare further aggravates the burdens of poor groups [2]. People who live in poor or marginalized communities with a low socioeconomic status have high chronic non-communicable diseases (NCDs) morbidity and mortality [13]. Since economic activity requires human capital, the combined eradication of poverty and improved human health has become a common human mission, with the United Nations’ 2030 Agenda for Sustainable Development, for example, putting forward targets to eradicate poverty and ensure healthy lives [2].

The effects of poverty, on the other hand, are generally concerned with living conditions, which in turn, influence people’s socio-economic status. Poor households were less able to cope with any given level of health expenditure than richer households [11]. Poverty exposes people to behavioral risk factors for NCDs [14], and the poor face disproportionate exposure to various NCD risk factors, which reduces access to critical prevention and treatment services. The poor often behave in less capable ways and suffer from a disproportionate burden of disease, yet usually have less access to health care [8, 15, 16]. Up to now, extensive studies have focused on the health-poverty nexus [2, 4], disease-driven poverty trap [17, 18], poverty and ill-health vicious cycle [19], ecosystem-poverty-health interaction [20] and medical poverty trap [21]. However, most studies only explore the relationship between poverty and ill-health, few reveal the health-income-place nexus of the poor.

China was once the country with the largest number of poor people in the world, and its public health problems have attracted worldwide attention. It has been engaged in improving human health and alleviating poverty since the 1980s. Great attempts have also been made to improve human health in rural areas, especially by reforming the health-care and health insurance system as well as implementing the health poverty alleviation project [12, 22, 23]. However, China’s poverty problems remain serious and become increasingly difficult to solve by conventional anti-poverty measures. Poverty-stricken people in China are gradually gathering in the deep rock mountains [24]. Not only is the current reimbursement scheme overly cumbersome, the effective reimbursement ratio is relatively low because of the high deductible, the low reimbursement ceiling, as well as medical expenditure outside the insurance catalogues [25, 26]. This because it fails to address expensive outpatient services, leading to high out-of-pocket (OOP) payments [25, 27].

Most previous studies examine the relationship between income/consumption poverty and such key health status indicators as mortality, morbidity and nutrition of individual health. In China, poverty alleviation usually targets household rather than individuals, and few studies quantify the relationship between poverty and ill-health in groups of poor people. In 2014, China has still 70.17 million people in the countryside living below the country’s poverty line of 2300 CNY ($376), of which 42% of them became impoverished because of illness and more than 14% of them are suffering chronic or serious diseases. Although the number of rural poor due to illness decreased from 28.5 million in 2014 to 5.16 million at the end of 2018, the proportion of poor due to illness remained about 40%. Therefore, it is vital and necessary to understand the health status of the rural poor in China and the mechanism of how the poor lead the farmers into poverty. To this end, based on nationwide health and socioeconomic survey data for poor households, this study investigated the cause of poverty, health status of poor households and assessed the impact of ill-health to farmers’ income in rural China. This study is a new and systematic attempt to understand the health status of the rural poor in China. Our findings would provide a scientific basis for future anti-poverty and health improvement strategies in China and beyond.

## Materials and methods

### Data sources

The data used in this study are from stratified random sampling questionnaire survey of 1223 villages in central and western provinces, which was conducted in 2017. The sampling of counties and villages considers the number and geographical location of the poor population, and adopts the random stratified sampling method to determine the survey counties and villages under the premise that the confidence interval is 95% and the sampling error is not less than 5%. The sample survey covers China’s 29,712 households.

The target population of the survey is poor households, which have been registered in the National Poverty Alleviation Information System of China since 2014. China has created a national registration system for the poor households that enables local official to gather data from each individual person, household and village [28]. The criteria for inclusion are having difficulty in stable access to food and clothing, or nine-year compulsory education, or basic medical services and housing, or an average annual net income per household below the national poverty line (CNY 2300 in 2014; CNY 1 = USD 0.153). One householder or sensible person for each household was interviewed in person. The questionnaire was designed to understand the basic situation, health status, employment, income status and poverty causes of poor households, as well as the longitude, latitude and elevation information of their residence.

### Methodology

#### Definition of variables

According to the definitions of the WHO and the Medium- to Long-term Plan on the Prevention and Treatment of Chronic Diseases in China (2017–2025), the types of illness in China are divided into NCDs and communicable diseases (CDs). In this study, the NCDs mainly include tumors, cardiovascular diseases, joint diseases, cerebrovascular diseases, chronic respiratory diseases, nervous system diseases, and endocrine/metabolic diseases. The types of disability are those defined by the PRC Disabled Protection Law as physical, mental, visual, intellectual, hearing, language and multiple disability. In the process of field survey, the income of farmers mainly includes four parts, namely, production and operation income, wage income, property income and transfer income. Among them, the income from production and operation includes the income from planting, breeding, service and processing industries. The net income of production and operation is obtained by subtracting various expenditures from the total income of production and operation. The property income includes land circulation fund, land equity dividend, micro credit equity dividend and village collective photovoltaic income dividend. Wage income is the income that farmers get from their work. Transfer income refers to all kinds of transfer payments made by the state and social organizations to households, including family planning subsidy, low insurance fund, special poverty support fund, endowment insurance fund and ecological compensation fund. In addition to the above subsidies, it also includes pension, social relief and subsidies, disaster relief funds, regular donations and compensation transferred by the government, non-administrative institutions and social organizations to farmers. Transfer income is an important part of the income source of Chinese farmers, especially for the poor groups who have no ability to work because of illness and disability. In addition, the longitude, latitude and elevation information of farmers’ residence are also collected. The variables and data sources involved in this study are shown in Table 1.

**Table 1:** Variables and data sources involved in this study

Variable	Definition	Sources
Poor households	The households with relatively difficult life. Their per capita net income is lower than the national poverty alleviation standard, and there are difficulties in eating and dressing, or their housing safety, basic medical care and compulsory education are not guaranteed.	Field investigation
Causes of poverty	The causes of farmers’ poverty	Field investigation
*Per_GDP*	The total income of rural households from various sources in the current year after deducting the expenses incurred	Field investigation
*AGE60*	Number of family members aged 60 and over	Field investigation
*WCP*	The number of people who have the ability to work from 16 to 60 years old	Field investigation
*ND*	The number of disabled family members	Field investigation
*N_NCDs*	The number of chronic patients in family members	Field investigation
*N_CDs*	The number of patients with infectious diseases in family members	Field investigation
*Elv*	The elevation of the residence of the farmer’s house	Field investigation

#### Correlation analysis

Pearson correlation analysis was used to explore the relationship between the family per capita net income, the altitude of the residence of the farmer and the number of the elderly aged 60 and over, the number of labor force (16–60), the number of disabled people, the number of chronic patients and the number of infectious patients, resulting in 49 correlations. The reported *p*-values were adjusted for multiple comparisons. The correlation between the two variables only means passing the significance test at least at 90% confidence level. Furthermore, the partial correlation analysis was applied to further reveal the relationship between the per capita net income of the family and the number of the family labor force, the number of the elderly, the number of the disabled, the number of chronic patients and the number of infectious diseases under the premise of keeping the geographical location unchanged. In the partial correlation analysis, the altitude of farmers’ residence is regarded as the condition control variable.

#### Regression analysis

Based on correlation analysis, the multiple regression analysis was further used to describe the quantitative relationship between the household’s health status and income. In the multiple regression model of this study, the dependent variable is the per capita GDP of the family (*Per_GDP*). The dependent variables include elevation (*Elv*), population aged 60 and over (*AGE60*), population with labor ability (*WCP*), population with disability (*ND*), population with chronic disease (*N_NCDs*) and population with infectious disease (*N_NDs*). The multivariable regression models using least squares approach were fitted under each of the scenarios representing the correlation matrix and dataset replications using the generated datasets with response variable *y* and predictors *x*_1_, *x*_2_, *x*_3_, *x*_4_, *x*_5_ and *x*_6_. The multiple regression model can be expressed as follows:

$$ Y={\upbeta}_0+{\upbeta}_1{x}_1+{\upbeta}_2{x}_2+{\upbeta}_3{x}_3+{\upbeta}_4{x}_4+{\upbeta}_5{x}_5+\varepsilon $$

Where β_0_, β_1_, β_2_, β_3_, β_4_ and β_5_ are the regression coefficients and the error term *ε* is normally distributed with mean 0 and variance σ^2^ (ε ∼ N(0,σ^2^)). Y, *x*_1_, *x*_2_, *x*_3_, *x*_4_, *x*_4_ and *x*_5_ are the *Per_GDP*, *Elv*, *AGE60*, *WCP*, *ND*, *N_NCDs* and *N_NDs*, respectively.

Additionally, the multicollinearity among the explanatory variables in the models was tested by the variance inflation factors (VIF). The VIF values are all less than 10, indicating no multicollinearity.

## Results

### Socio-economic and demographic characteristics of farmers

Most of the respondents are male, and their education level is generally low. Of the 29,712 respondents, 80% of them were male, and 95% were illiterate or semi illiterate, and 21% were only primary school educated. The average household size of the respondents was 6, including 11.8% for single person, 22.4% for two persons, 19.8% for three persons, 21.7% for four persons, 13.16% for five persons and 7.6% for six persons per family. The aging characteristics of the poor are outstanding. Among the respondents, 2480, 3150 and 62 have only one, two and three people, all of whom are aged 60 and above. The respondents’ family income level was low, the source was single, income increase was difficult, and they were highly dependent on the government’s transfer income. Of the 29,712 families interviewed, 82 had much more productive and operational expenditure than their income, and they were in a state of not making ends meet, and the per capita net income of 21,055 families was less than 3000 yuan. In addition, 1919 families’ income only comes from the government’s transfer income.

### Causes of poverty

**Fig. 2:**
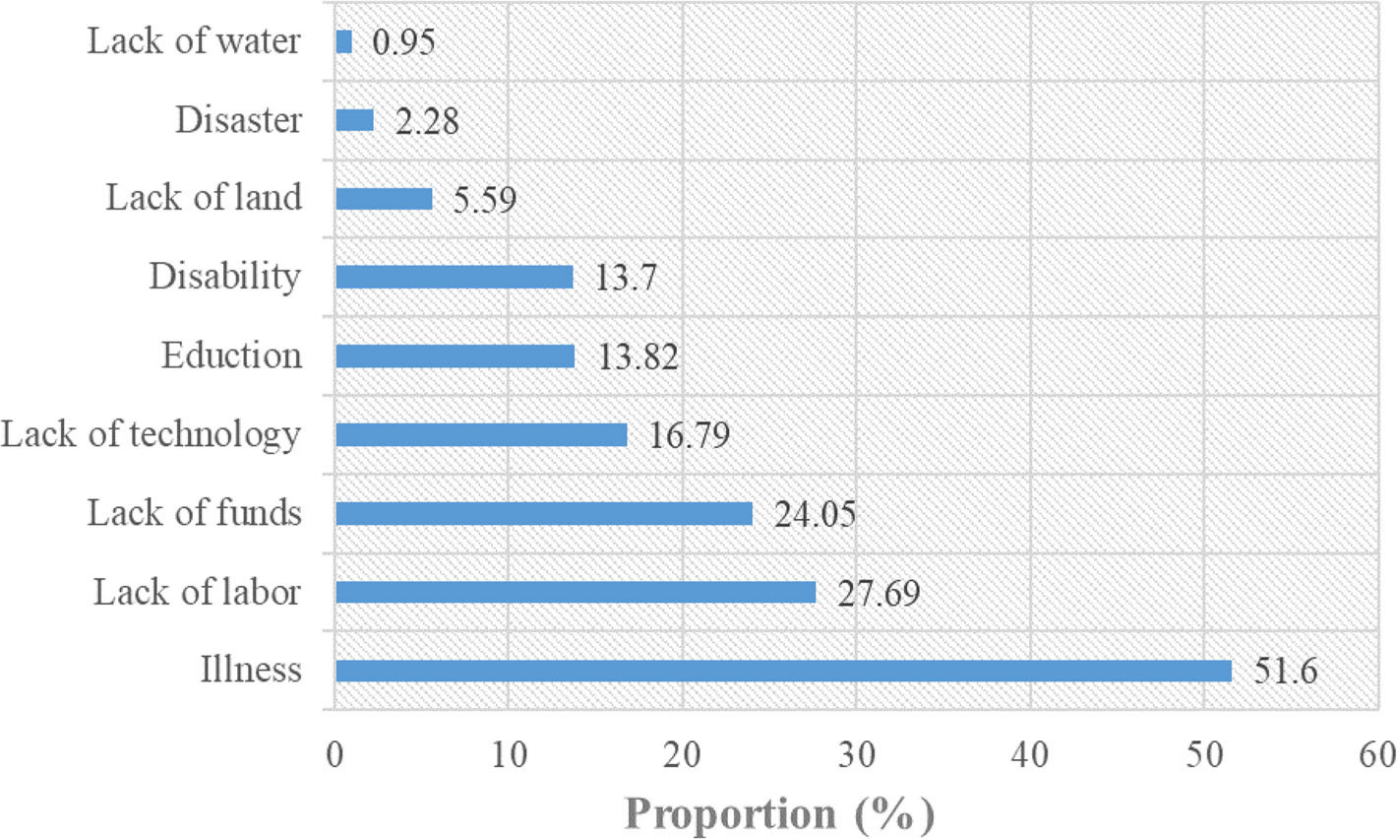
Causes of poverty for the interviewed households

Understanding the causes of poverty at the household level is a vital step toward combating poverty around the world. Figure 2 gives the major causes of poverty for the surveyed households, indicating illness to be the biggest cause of poverty in rural China. More than half of the households in our survey attribute their poverty to household members suffering from diseases. Approximately 14% of the households ascribed their poverty to high educational costs or disabilities of household members and 2.28% to natural disasters. In addition, the lack of farmland and water resources were also important driving forces for farmers’ poverty. Obviously, illness is the main root of poverty in most low-income groups in rural China. Poverty is a multidimensional issue, but the lack of self-development capacity due to illness is a significant contributor to the poverty of rural households.

### Health status

**Fig. 3:**
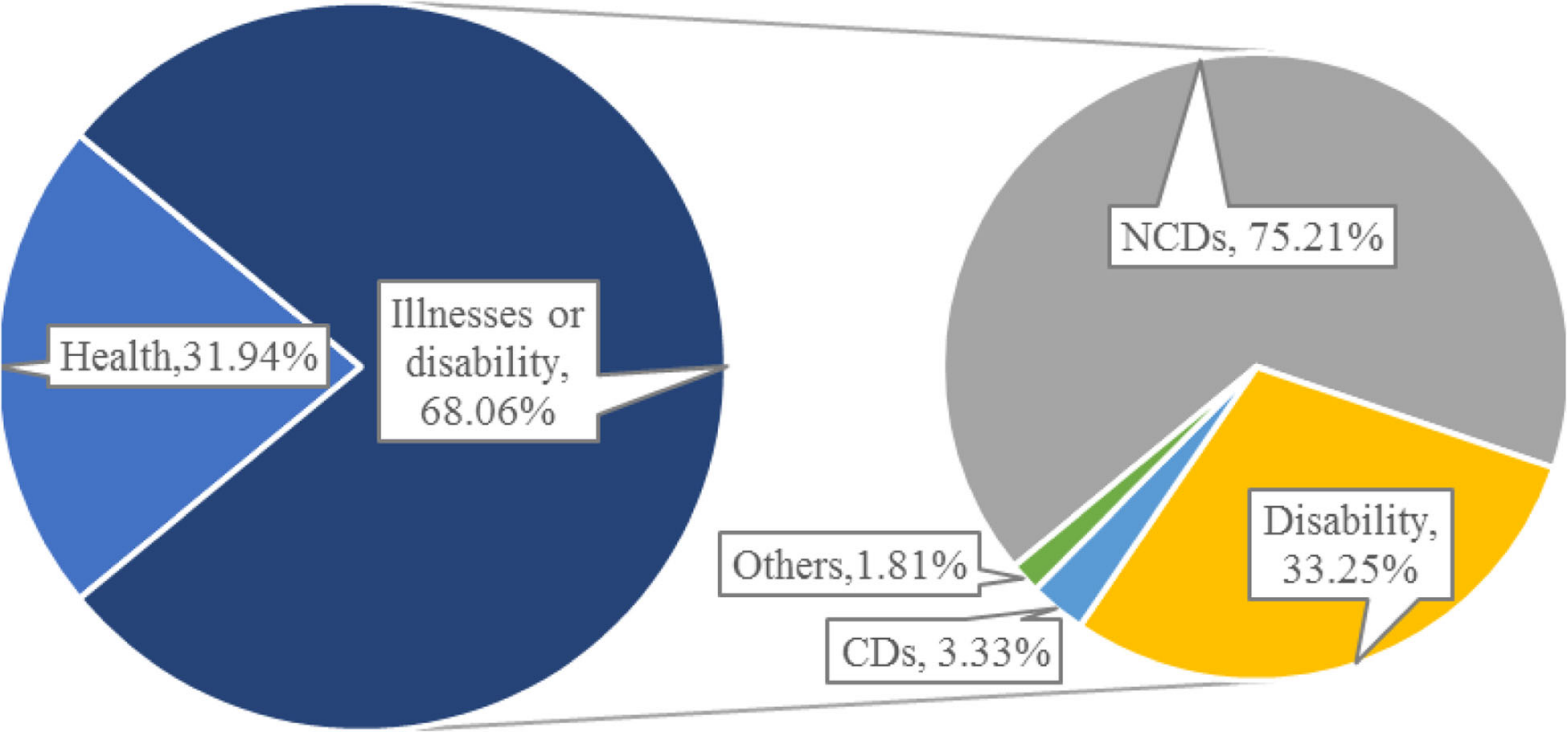
Health status of the surveyed household members (Notes: In unhealthy families, a family may have both chronic or infectious patients and disabled people, so the proportion in the right figure is more than 100%.)

The health status of poor rural households in China is not optimistic. Chronic diseases are the main disease types. It is common that minor diseases are not treated for a long time. Figure 3 shows the health status of the households, with 60.08% having at least one member who is a disease or disability patient, three quarters of which (75.21%) involve NCDs, and a third (33.25%) disabled.^1^ Further statistics show that 8.12% of the households had both NCD and disabled patients, 0.96% both NCD and CD patients, and 0.30% both CD and disabled patients. What is worse, 41 households had the NCD, CD and disabled patients at the same time. The proportions of the families with one, two, three, four, five, six and seven kinds of diseases were 42.5, 17.78, 5.81, 1.62, 0.27, 0.07 and 0.02%, respectively.

#### Non-infectious chronic diseases (NCDs)

**Fig. 4:**
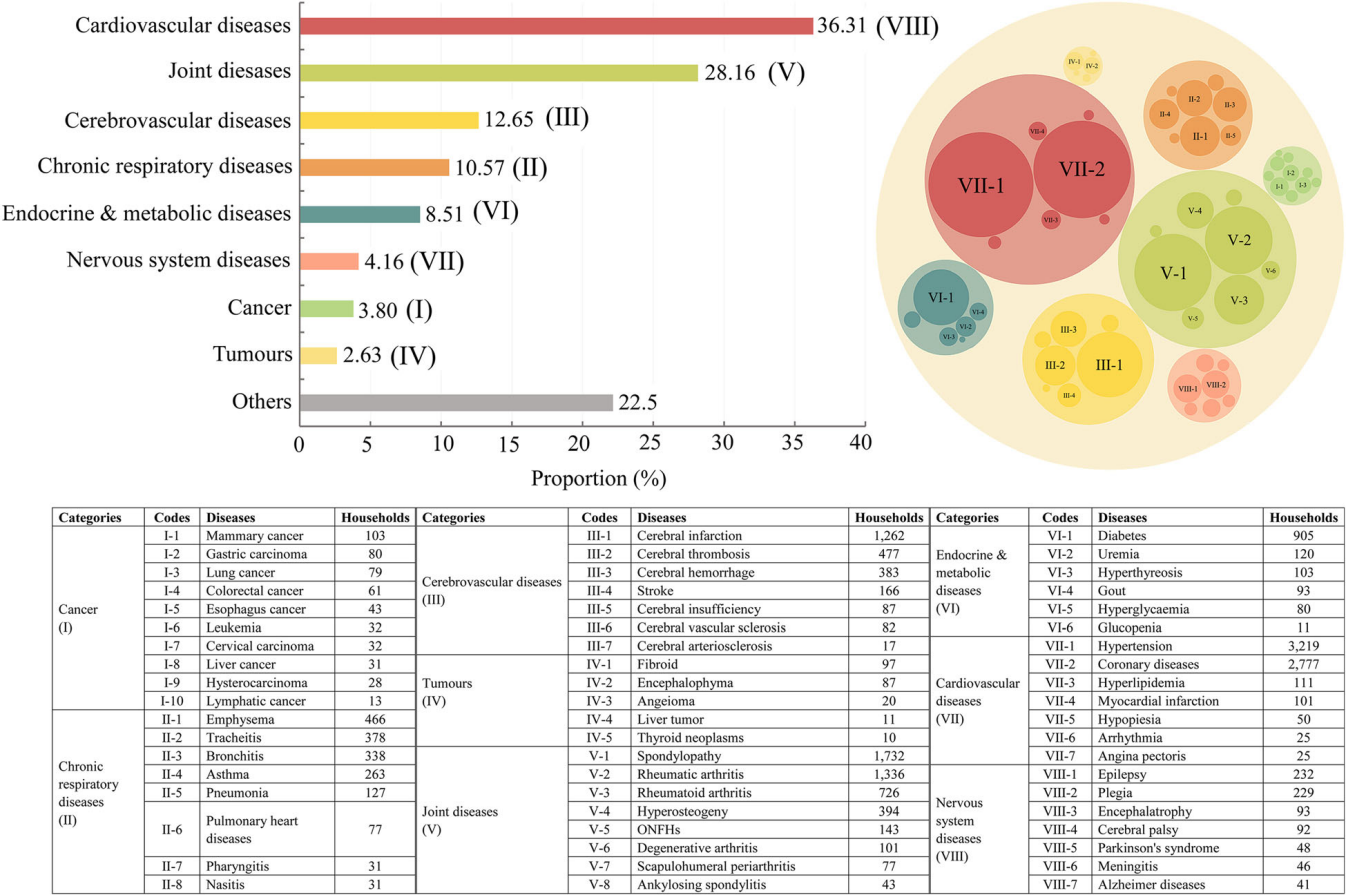
Types of NCDs

As Fig. 4 indicates, the households have patients with many kinds of NCDs, mainly tumors, cancers, cardiovascular disease (CVD), chronic cerebrovascular disease (CCD), bone and joint disease (BJD), nervous system disease (NSD), chronic respiratory disease (CRD), and endocrine and metabolic disease (EMD). Of each NCD household, an average of 1.47 members are chronic. More than one-third of the households suffered from CVD. The proportion of the surveyed families suffering from BJD, CCD, CRD, EMD, cancer, and tumor was 28.16, 12.65, 11, 9, 3.8, and 2.63%, respectively.

Specially, the cancer in the survey included mammary cancer, gastric carcinoma, lung cancer, colorectal cancer, esophagus cancer, leukemia, cervical carcinoma, liver cancer, hysterocarcinoma and lymphatic cancer. The most prevalent cancers are mammary cancer, gastric carcinoma, lung cancer, colorectal cancer and esophagus cancer, while the most prevalent tumors are uterine myoma, brain tumor (encephalophyma), angeioma, liver tumor and thyroid neoplasms. In the households with cancer patients, 20.6% of them suffered from breast cancer, 15.94% from stomach cancer and 15.73% from lung cancer. In the households with tumor patients, 24.25, 21.75, 5, 2.75 and 2.5% of them suffered from uterine myoma, brain tumor, angeioma, liver tumor and thyroid neoplasms.

Chronic cerebrovascular disorders (CCD) is also a major chronic disease, accounting for nearly 50% of the NCD households. The incidence of cerebral infarction is the highest in the CCD households, accounting for 65%. Families with CVD patients were even more common than those with CCD patients and the former was 2.9 times as much as the latter. Hypertension and coronary heart disease are the most common CVD. Of these, 58.3% have hypertension and 50% coronary atherosclerotic heart disease. More than 76% of the households with hypertensive patients had at least one member over 60 years old. In addition, some surveyed households also suffered from other cardiovascular diseases, such as hyperlipidemia, myocardial infraction and arrhythmia. Cerebral thrombosis and cerebral hemorrhage are also common cerebrovascular diseases, accounting for 24.8 and 19.9% of the CCD households. The incidence of stroke is also relatively high, representing a threat to human health as serious as coronary heart disease and cancer.

Bone and joint disease (BJD) is also a common chronic disease that has plagued Chinese rural residents for a long time. Around 28.16% of similar NCDs households have BJD, with vertebral disease being the most prevalent at over 40% of the BJD households. Rheumatic and rheumatoid arthritis also account for 48.12% of the similar diseases. Arthritis occurred mainly among the middle-aged and elderly people. Hyperosteogency and osteonecrosis of the femeral head, at 12.5%, are relatively common.

Chronic respiratory disease (CRD) is characterized by high morbidity, high disability rate and heavy burden of disease, which has become one of the prominent public health problems in China [29]. The incidence of the CRD is also relatively high, next only to CCVD and BJD, with over 5.39% households having CRD patients, for whom chronic obstructive pulmonary disease (COPD, including emphysema and chronic bronchitis) is the most common, followed by bronchial asthma, chronic cor pulmonale and chronic pneumonia. As the population ages, smoking, air pollution, new pathogens and drug-resistant pathogens, the prevention and control of the CRD is becoming increasingly more challenging [30].

Endocrine and metabolic disease (EMD), including diabetes, uremia, gout (hyperuricemia), hyperglycemia and hyperthyreosise, is also a common chronic disease that bothers rural residents, with over 3% of households having diabetes. Diabetes has become the world’s third chronic disease after tumor and cardio-cerebrovascular diseases and seriously threatened human health. Importantly, morbidity crowd of diabetes is becoming younger. Such other nervous system diseases as epilepsy, encephalatrophy, Parkinson’s syndrome and Alzheimer disease are also prevalent.

#### Communicable diseases and disability

The households have such communicable diseases as pulmonary tuberculosis, viral hepatitis, brucellosis, poliomyelitis, encephalitis, tuberculous pleurisy and AIDS. Of these, tuberculosis is the most prevalent, followed by hepatitis and poliomyelitis. Most surprisingly, some in the northeastern part of the Tibetan Plateau have brucellosis (also known as caprine and ovine brucellosis, an infectious disease through sheep), affecting 22 herdsman household members in Qinghai province. Since brucellosis is an endemic disease, it should be given high attention by relevant departments. Both the high burdens of endemic disease and the unanticipated effects of disease control may act as poverty traps. The effective control of these diseases still requires effective public health infrastructures to rapidly recognize and respond to them [31].

The loss of labor force or deprivation of basic capabilities caused by household member’s disability [32]. There were seven types of disability among the surveyed households, i.e. physical, mental, visual, intellectual, hearing, language and multiple disability. Physical disabilities are quite common and account for 50.47% of the disabled households, followed by mental, visual and intellectual disability. More than 20.37% of households have two or more patients with disabilities.

#### Medical security system

More than a quarter of the households with diseased or disabled patients cannot afford expensive medical expenses, nearly half of which have no health insurance. Although 98% of households involved in the New Cooperative Medical Scheme (NCMS), among which 16% of them are still unable to bear their household medical expenses after reimbursement. In addition, the recourse for many is private (folk or barefoot) doctors or itinerant medicine sellers since public hospital treatment is ineffective, the cost of which is not within the scope of the NCMS reimbursement - increasing OOP expenditure.

### The nexus between ill-health and poverty

Table 2 provides the descriptive statistical results of variables. Of the 29,712 households surveyed, the average annual net income of households was 5870 CNY (around $851). In terms of health status, an average of one quarter of the households surveyed had disabled persons and three quarters had chronic disease patients. The number of families with infectious diseases is relatively small. The average elevation of the surveyed households’ residence was over 830 m. This indicates that at present, most of China’s poverty-stricken population is concentrated in the deep mountainous areas.

**Table 2:** Descriptive statistics

Variable	Average value	Standard deviation	Sample size
*Per_GDP*	5870.71	5721.70	29,712
*AGE60*	0.79	0.82	29,712
*WCP*	0.55	0.78	29,712
*ND*	0.25	0.49	29,712
*N_NCDs*	0.75	0.91	29,712
*N_CDs*	0.02	0.15	29,712
*Elv*	830.15	888.17	29,712

Notes: *Per GDP* is per capita GDP; *AGE60* is the number of household members aged 60 and over; *WCP* is the number of household members with working capacity; *ND, N_NCDs* and *N_CDs* are the number of households affected by disability, *NCDs* and *CDs* respectively; and *Elv* is the elevation of the interviewed households’ residence

The poverty-health-place nexus is examined by the Pearson and partial correlation analysis. Results show that the per capita net income of the households surveyed was positively correlated with the number of working abilities, negatively correlated with the number of elderly populations, chronic patients, infectious patients and disabled persons and its altitude. The higher net income per capita of some families at higher altitudes may mean that these families receive more government transfer income. There was a significant negative correlation between the elevation of the interviewed farmers’ residence and the number of chronic patients. The number of the elderly families was significantly positively correlated with the number of families with chronic infectious diseases. This means that the more people aged 60 or older in the family interviewed, the more likely they were to suffer from chronic diseases. If the elevation was taken as a control variable, the per capita net income was still positively correlated with the number of labor force, and negatively correlated with the aging rate, the number of disabled persons, the number of chronic patients and the number of infectious diseases. But the correlation coefficients between them are all reduced. This result demonstrates that the impact of unhealth on income will be weakened with the elevation of farmers’ residence (Table 3).

**Table 3:** Partial correlation analysis results

Control variable	Variable	*Per_GDP*	*AGE60*	*WCP*	*ND*	*N_NCDs*	*N_CDs*	Elv
No	*Per_GDP*	1	− 0.12***	0.20***	−0.05***	− 0.12***	− 0.02***	− 0.02***
	*AGE60*	−0.10***	1	−0.09***	0.05***	0.25***	0.001	−0.14***
	*WCP*	0.20***	−0.10***	1	−0.07***	− 0.07	− 0.04***	0.09***
	*ND*	−0.05***	0.05***	−0.07***	1	−0.14***	− 0.03***	− 0.05***
	*N_NCDs*	− 0.12***	0.25***	− 0.07***	− 0.14***	1	− 0.02***	− 0.14***
	*N_CDs*	−0.02***	0.001	−0.04	− 0.03***	− 0.02***	1	− 0.01*
	*Elv*	−0.02***	− 0.14***	0.09***	− 0.05***	− 0.14***	− 0.010*	1
Elv	*Per_GDP*	1	−0.10***	0.20***	−0.05***	−0.15***	− 0.02***	
	*AGE60*	−0.10***	1	−0.08***	0.04***	0.24***	0	
	*WCP*	0.20***	−0.08***	1	−0.07***	−0.06***	− 0.003	
	*ND*	−0.05***	0.04***	−0.07***	1	−0.15***	−0.03***	
	*N_NCDs*	−0.12***	0.24***	−0.06***	−0.15***	1	−0.02***	
	*N_CDs*	−0.02***	0	0	−0.03***	−0.02***	1	

Notes: *Per GDP* is per capita GDP; *AGE60* is the number of household members aged 60 and over; *WCP* is the number of household members with working capacity; *ND*, *N_NCDs* and *N_CDs* are the number of households affected by disability, *NCDs* and *CDs* respectively; and *Elv* is the elevation of the interviewed households’ residence. The sample size is 29,712. * Indicate statistical significance at the 10% level. *** Indicate statistical significance at the 1% level

The multiple regression results further confirm the health status of households is highly correlated with their income (Table 4). The more diseases a household has, the less income it gets. For every increase in the number of a household suffering from chronic disease, infectious disease and disabled patient, per capita GDP tend to decrease by CNY 615, 854 and 569, respectively. Moreover, the family with one more elderly tend to decrease its per capita GDP by CNY 419, and the family with one more labour force tend to increase its per capita GDP by CNY 1386. The elevation of peasant households is negatively correlated with their per capita net income, but the correlation coefficient is not high. For every 1000 m increase in altitude, net income per capita will decrease by CNY 120.

**Table 4:** Summary statistics for the regression models between health, per capita income and geographical location

Variable	Unstandardized Coefficients		Standardized Coefficients	t	Sig.	Collinearity Statistics	
	B	Std. Error	Beta			Tolerance	VIF
(Constant)	6167	69.96		88.15	0.000		
*Elv*	−0.120	0.037	− 0.019	−3.234	0.001	0.960	1.041
*AGE60*	− 419.855	40.930	−0.060	−10.258	0.000	0.914	1.094
*WCP*	1386.370	41.950	0.188	33.048	0.000	0.979	1.021
*ND*	− 569.758	67.053	−0.049	−8/497	0.000	0.963	1.038
*N_NCDs*	− 615.258	37.472	−0.098	−16.419	0.000	0.897	1.115
*N_CDs*	− 854.289	213.857	−0.023	−3.995	0.000	0.998	1.002
Adjusted *R*^2^	0.058						
F(*p*)	305.598 (0.00)						

Notes: Dependent variable: Per_GDP; Independent variable: Elv, AGE60, WCP, ND N_NCDs, N_CDs. Per_GDP is per capita GDP; Elv is the elevation of the interviewed households; AGE60 is the number of household members aged 60 and over; WCP is the number of household members with working capacity; ND, N_NCDs and N_CDs are the number of households affected by disability, NCDs and CDs, respectively. The sample size is 29,712

## Discussion

This study investigated the cause of poverty and health status of 29,712 households from China’s 117 counties and explored the relationship between poverty and health. We found illness to be the biggest cause of poverty in rural China. More than half of the households attribute their poverty to household members suffering from diseases, higher than the national average level (44.1%) [33]. The higher proportion of the households suffered from diseases may be since our survey mainly concentrated in the poorest regions of the country. According to a national survey, for the impoverished households with at least one patient (i.e., diseased or disabled member), there were 3.3 million people suffering from serious illness and 4 million people from chronic diseases, of whom the patients aged 15–59 years old accounted for 41% [34].

Cancer incidence and mortality have been increasing in China with more than 16 million people being diagnosed and 12 million people dying each year [35], making cancer the leading cause of death since 2010 [36]. The most prevalent cancers in the survey are mammary cancer, gastric carcinoma, lung cancer, colorectal cancer and esophagus cancer, while the most prevalent tumors are uterine myoma, brain tumor (encephalophyma), angeioma, liver tumor and thyroid neoplasms). These findings are accord with previous studies that also found that lung, stomach, liver and esophageal cancer to the four most common cancers diagnosed, which accounted for 57% of cancers diagnosed in China and one-half of the global incidence burden [36]. Rural poor groups have higher age-standardized incidence and mortality rates for cancers than urban residents because of higher smoking prevalence and limited medical resources [10]. Thus, reducing the prevalence of risk factors and increasing the effectiveness of clinical care delivery for rural poor populations may help to reduce the incidence and mortality of cancer [36].

Poverty and ill-health mutually influence. Ill-health is a major contributor of poverty and it may prolong the duration of impoverishment. NCDs have a large negative impact on productive capacity and become important drivers of poverty [14], which can expand and deepen poverty through catastrophic out-of-pocket (OOP) medical expenditure, by forcing people to exit the labor market, and/or by forcing children out of school to act as caregivers [10]. The NCDs is prevalent among the poor [9]. Our survey suggests that current medical security system is insufficient to effectively reduce the medical burden of rural poor households in China. Meanwhile, health insurance coverage for the poor is inadequate. Although the reimbursement mechanism of medical insurance system can help to reduce farmers’ OOP payment, its effect is still limited and needs to be improved [37]. Since 2003, China has implemented the NCMS, a public health insurance program in rural China, to reduce the medical burden of farmers. The proportion of the OOP health expenditure to total health expenditure for rural residents in China has decreased from 46.4% in 1995 to 32% in 2014, but it is still above the global average level of 18.17% [38]. OOP health payments remain a severe burden for rural households [39]. In rural China, private health expenditures comprised about half of total health expenditures and most of these expenditures were OOP. These results indicated that social health insurance participation can increase the enrollees’ access to medical service, but it did not effectively lower the OOP payment [27]. Fortunately, to further reduce the medical burden of the poor, the country has started to implement the health poverty alleviation project since 2017, and implemented disease classification treatment for the diseases (serious, chronic and serious) of the rural poor. It is required that all the poverty-stricken people in rural areas should be covered by basic medical insurance, serious illness insurance and medical assistance. This country also strived to improve the level of medical and health facilities and talents in poverty-stricken areas. It requires at least one county-level public hospital in each poverty-stricken county, one standardized township health office in each township, and one health office in each administrative village. At the beginning of 2018, a telephone survey on satisfaction of health poverty alleviation conducted by the national health department in China showed that 83.9% of the poor people in the country were satisfied with the implementation of health poverty alleviation at the bottom [40]. These measures have greatly reduced the medical burden of the poor, and improved the medical and health facilities and service level in the poor areas.

Revealing the connections between poverty and health conditions can help to address both the causes and consequence of the diseases in the long term. Our results mean that the economic impact of ill-health on rural low-income households can be substantial, which creates a vicious cycle that forces people into poverty and more illness. On the contrary, high-income earners could afford some level of protection against infectious diseases through nutrition and better sanitary conditions [2]. Usually, per capita income falls as diseases prevalence rises because economic activities require healthy physical conditions. While health improvement is a prerequisite to economic growth and poverty reduction, disease-led poverty is becoming increasingly obvious, and the internal impetus for economic growth in impoverished areas is said to be insufficient. Health improvement and poverty reduction are interlinked and mutually reinforcing. Effective control of the diseases involved still requires effective public health infrastructures to rapidly recognize and respond to them [32]. Early childhood interventions can substantially boost adult health and wage earnings [41], further blocking the intergenerational transmission of poverty. It is necessary to further decrease the OOP payments by decreasing or abolishing deductible, expanding the drug list and check directory of China’s NCMS benefit package and increasing the reimbursement ratio of outpatient services to minimize the gap between the nominal reimbursement ratio and effective reimbursement ratio [25–27]. Extending access to affordable and high-quality care is urgent to avoid the medical poverty trap. These measures can contribute to get access to affordable and high-quality care and avoid the medical poverty trap, thus reducing the prevalence of risk factors and increasing the effectiveness of clinical care delivery for rural poor populations may help to reduce the incidence and mortality of cancer.

## Conclusions

Based on the data from nationwide cross-sectional survey, our analysis indicates that illness is the major cause of rural poverty in China. More than half of the rural poor households in this survey attributed their poverty to household members suffering from diseases. Meanwhile, illness usually leads to a lack of working ability. Our survey showed that more than a quarter of the households attributed their poverty to the lack of labor. Moreover, lack of land and water resources are also important driving forces of rural poverty. These findings demonstrate that poverty is a multidimensional problem and the shortage of endogenous impetus is an important influence. Further findings showed that the health status of the rural poor in China is not optimistic. More than 60% of households have members with various diseases or disabilities. NCDs are very common and 75.21% have NCD patients. A household may have more than one patient and each patient may have more than one disease and/or disabilities. Approximately 10% of households contain both NCD and disabled patients, with a small number having both NCDs, CDs and disabilities. Although China has made great efforts to improve the medical service level and medical security system in poor areas in recent years, the poor still face a great burden of medical care. More than 98% of the interviewed households are involved in NCMS, but 16% of these are still unable to bear their medical expenses after reimbursement.

Furthermore, geographical position, ill-health and poverty are interlinked and mutually reinforcing. The elevation of farmer’s residence affects medical infrastructure or services, thus affecting human health and family income. The higher the altitude of the farmer’s residence, the lower the level of medical services they enjoy, and the worse their health. Therefore, there is a significant negative correlation between the altitude of the farmer’s residence and the health status. The ill-health status of households is also significantly negatively correlated with per capita net income. Both chronic and infectious diseases have a larger negative influence than disability on household income, with per capita GDP decreasing by CNY 615~854 for every increase in the number of diseases (including chronic and infectious diseases) and by CNY 569 for every increase in the number of disabled patients. This result indicates that compared with the disability, both chronic and infectious diseases impose larger negative influences on farmers’ income.

Although recent decades have seen remarkable achievements in poverty alleviation and health improvements in China, the rural poor groups who are poor due to illness and return to poverty due to illness have remained at about 40% at the end of 2018. Further measures are needed to achieve the goal of health China 2030 and rural revitalization 2050. First, effective goal-oriented and targeted pro-poor health interventions - involving local, national, global, public and private initiatives - are required that prioritize and respond to the needs of poor people. This includes expanding health insurance coverage, decreasing illness insurance deductibles, lowering the OOP expenditure and raising the actual reimbursement ratio through critical illness insurance; linking and combining multiple insurance systems to improve the level of healthcare security for the poor, and basic healthcare insurance and additional subsidies for individuals with NCDs and critical illnesses to cover their (currently unaffordable) basic medical expenses. Encouraging a healthier lifestyle to reduce exposure to tobacco, alcohol, unhealthy diet and physical inactivity is also important, since it can contribute to improve cognitive capacities and learning outcomes, and stimulate the endogenous impetus of poor groups, which is beneficial for the human capital of rural economic development. Further investigation of the medical expenditure for poor groups and their access to health care resources would help to provide a strong justification for more government-funded health resources and services for disease control in poverty-stricken areas. Second, considering endemic diseases and their pathogenesis in the prevention and control of diseases is essential for improving the health of the poor. Third, more policies are urgently needed to focus on the health conditions of the elderly as many older rural people with multiple chronic diseases (i.e., hypertension, hyperlipidemia, heart disease, diabetes, stroke and ischemic cerebrovascular disease). Last, accelerating the establishment of an urban-rural integrated medical security system with a health improvement policy should be pay equal attention to both the rural and urban poor. Overall, given the importance of the contribution of China to global poverty reduction and health improvement, implementing targeted and goal-oriented health interventions for poor groups would have a great impact on global poverty eradication and sustainable development.

## Data Availability

General correspondence and requests for source data and materials should be addressed to Y. Zhou. Requests for access to data should be addressed to Y. Zhou (zhouyang@igsnrr.ac.cn).

## Footnotes

1 *Notes*: The total effective sample size was 29,712. The sample size of all kinds of diseases is larger than the total sample size because an interviewed household may suffer from various diseases.
